# Targeting PDPN enhances antitumor T-cell activity by disrupting β-catenin-mediated PD-L1 expression in melanoma

**DOI:** 10.3389/fimmu.2025.1692864

**Published:** 2026-01-07

**Authors:** Chunyan Feng, Yueyue Liu, Xinyue Zhong, Zhenzhen Wei, Hongjing Cui, Jianfeng Yi

**Affiliations:** Research Center for the Differentiation and Development of Traditional Chinese Medicine Basic Theory, Institute for Advanced Study, Jiangxi University of Chinese Medicine, Nanchang, Jiangxi, China

**Keywords:** CD8 lymphocytes+, immunotherapy, PD-L1, podoplanin, Wnt/β-catenin

## Abstract

**Introduction:**

Melanoma is a highly immunogenic and aggressive malignancy characterized by pronounced intratumoral lymphocytic infiltration and significant responsiveness to immunotherapeutic interventions. The oncogenic glycoprotein podoplanin (PDPN) is commonly overexpressed in various cancer types, where it facilitates metastatic dissemination through interactions with CLEC-2 on platelets and other stromal cells, thereby contributing to stromal immunosuppression. Although the protumoral roles of PDPN are well documented, its precise mechanistic contributions to immune evasion in melanoma remain only partly defined and require further elucidation.

**Methods:**

To clarify the immunological role of PDPN in melanoma, multiplex immunofluorescence staining was performed on human tissue microarrays, and bioinformatic analyses were conducted to determine the associations of PDPN with PD-L1 expression and CD8^+^ T cell infiltration. The therapeutic efficacy and underlying mechanisms of the PDPN-targeting inhibitory peptide CY12-RP2 were systematically evaluated using flow cytometry, Western blotting, ELISA, and *in vivo* studies in both immunodeficient and immunocompetent mouse models. These comprehensive analyses demonstrated that targeting PDPN with CY12-RP2 can reestablish antitumor immunity.

**Results:**

Multi-omics analyses indicated that PDPN expression is highly correlated with immune checkpoint markers, most notably PD-L1 (r = 0.504, p < 0.001), and displays an inverse relationship with the infiltration of intratumoral cytotoxic immune cells. Single-cell and spatial transcriptomic profiling revealed that PDPN supports the exclusion of CD8^+^ T cells and enhances the prevalence of immunosuppressive cell populations. CY12-RP2 resulted in a 60.6% reduction in tumor growth in immunocompetent murine models and reversed immune evasion by attenuating PDPN-dependent, β-catenin-mediated upregulation of PD-L1. Treatment with CY12-RP2 broadly activated antitumor immune responses, as evidenced by increased intratumoral infiltration of CD8^+^ T cells, elevated granzyme B production by CD8^+^ T cells, and enhanced secretion of pro-inflammatory cytokines (IFN-γ, TNF-α, and IL-1β). Depletion experiments confirmed that the antitumor efficacy of CY12-RP2 was entirely dependent on CD8^+^ T cells, establishing a CD8^+^ T cell-dependent mechanism of action.

**Discussion:**

These findings identify PDPN as a critical driver of immune evasion in melanoma via b-catenin-mediated PD-L1 upregulation. Inhibitory targeting of PDPN with CY12-RP2 represents a promising therapeutic approach capable of disrupting this immunosuppressive pathway and reversing tumor immune escape.

## Graphical Abstract

**Figure:**
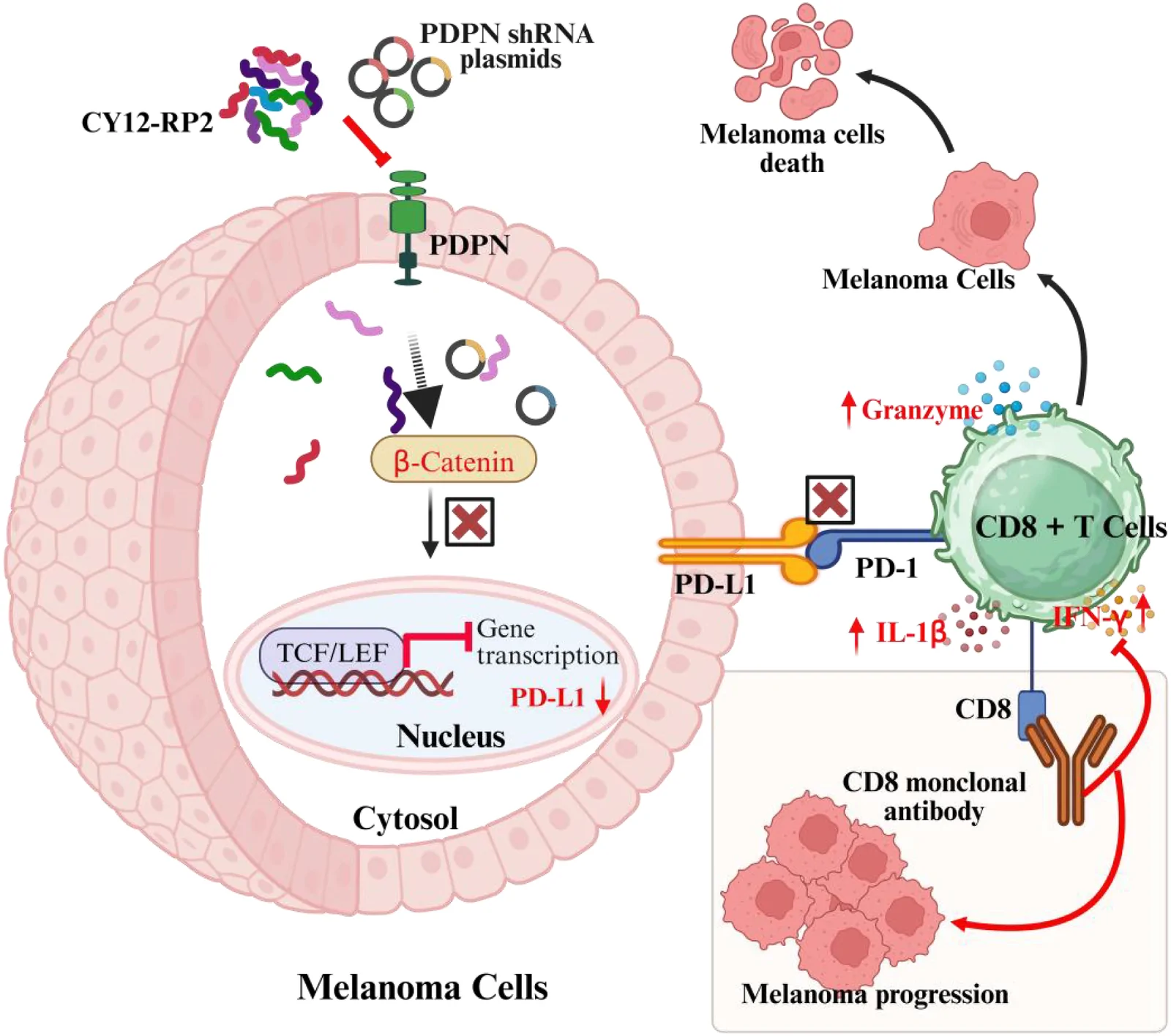

## Introduction

Melanoma is a highly immunogenic malignancy originating from melanocytes and is distinguished by a significantly higher propensity for metastasis compared to other cutaneous cancers (1, 2). This strong immunogenicity is evidenced by dense lymphocytic infiltration within the tumor microenvironment (TME) and an increased incidence among immunocompromised individuals (3, 4), rendering melanoma particularly amenable to immunotherapeutic strategies. While immunotherapies—such as immune checkpoint blockade and adoptive cell transfer—exhibit superior therapeutic indices and fewer off-target toxicities relative to conventional surgery, radiotherapy, or chemotherapy, their clinical application in melanoma has advanced more slowly than in breast, lung, or liver cancers. This slower pace is largely attributable to the absence of robust, melanoma-specific predictive biomarkers. Consequently, the identification of reliable biomarkers to guide patient stratification, optimize treatment selection, and inform rational design of combination immunotherapeutic approaches remains an urgent and critical objective in melanoma management. Advancing the discovery and clinical utilization of such markers is essential for realizing the full promise of immune-based therapies against this aggressive disease.

Podoplanin (PDPN) is a mucin-type transmembrane glycoprotein with pivotal roles in embryonic development and platelet aggregation (5). PDPN is aberrantly overexpressed across various malignancies—including hepatocellular carcinoma (6, 7), glioma (8, 9), breast cancer (10), and lung squamous cell carcinoma (11, 12)—where it actively drives tumor initiation, progression, and metastatic spread. Mechanistically, PDPN associates with CLEC-2 receptors on platelets via its extracellular PLAG3 domain, thereby facilitating platelet activation and promoting tumor cell invasion (13). Within tumor cells, PDPN interacts with the ERM protein family, contributing to epithelial–mesenchymal transition (14). Beyond direct tumor cell functions, PDPN also plays a critical role in shaping the immunosuppressive tumor microenvironment (TME) (5, 8, 15). In melanoma, expression of PDPN by intratumoral cancer-associated fibroblasts (CAFs) is correlated with increased sentinel lymph node metastases, with PDPN-expressing CAFs enhancing local immunosuppression through cytokine secretion and immune modulation (16, 17). Tumor cell-intrinsic PDPN impairs the cytotoxic activities of CD8^+^ T cells, natural killer (NK) cells, and macrophages, potentially through IL-27-dependent processes (5, 15). The PDPN–CLEC-2 axis further supports the recruitment of immunosuppressive macrophages, and pharmacologic blockade of this pathway reduces macrophage accumulation and alleviates local immune suppression (18). Additionally, previous studies, including our own, have demonstrated that the PDPN-targeting inhibitory peptide CY12-RP2 augments antitumor immunity by enhancing the infiltration and activation of cytotoxic CD8^+^ T cells, NK cells, and M1-polarized macrophages, with concurrent increases in pro-inflammatory cytokine secretion (e.g., IFN-γ, TNF-α) (19). To gain a comprehensive understanding of PDPN-driven immune evasion, we used the same tissue microarray (HMelC112CD01). This investigation is necessary to realize PDPN’s potential as a therapeutic target for improving antitumor immunity, especially in melanoma.

Tumor cells evade T cell-mediated immune surveillance primarily through upregulation of PD-L1 (20). Emerging evidence indicates that anti–PD-1 therapy can trigger adaptive upregulation of PD-L1, contributing to acquired resistance to immune checkpoint blockade (ICB) (21). Delineating the molecular mechanisms that control PD-L1 expression is therefore crucial for optimizing ICB efficacy and improving patient outcomes. The regulation of PD-L1 is multifactorial: extrinsic regulation is mediated by cytokines in the TME (such as TNF-α, IFN-γ, and IL-1β) (22), while intrinsic regulation involves oncogenic signaling pathways. Although several oncogenic drivers—including MYC, EGFR, and RAS—are established regulators of PD-L1 (23–25), the precise molecular mechanisms through which PDPN modulates PD-L1 remain incompletely defined.

The β-catenin signaling pathway represents a critical mechanism facilitating tumor immune evasion, primarily through its regulation of PD-L1 expression. In glioblastoma, activation of β-catenin directly induces transcription of CD274 (encoding PD-L1) via β-catenin/LEF1 binding to the CD274 promoter, a process that is maintained by AKT signaling and is associated with reduced intratumoral CD8^+^ T cell accumulation (26). In addition to direct transcriptional control, β-catenin signaling broadly suppresses antitumor immune responses in the TME by impairing T cell priming by dendritic cells (DCs) and fostering immune tolerance (27, 28). Melanoma model systems have demonstrated that activation of the β-catenin pathway leads to the exclusion of CD8 + T cells, conferring resistance to anti–PD-L1 therapy (29). Although phosphorylation of β-catenin at S552 facilitates its nuclear translocation, its specific role in regulating PD-L1 in melanoma and other tumors remains insufficiently characterized.

In the present study, we establish that inhibition of PDPN using the CY12-RP2 inhibitory peptide attenuates melanoma progression in multiple murine models, concomitant with reduced functional exhaustion of tumor-infiltrating CD8^+^ T cells. Mechanistically, CY12-RP2 acts by suppressing PDPN-mediated activation of the Wnt/β-catenin signaling cascade, resulting in diminished PD-L1 expression in melanoma cells and subsequent activation of CD8^+^ T cell-mediated antitumor responses. While PDPN has been identified as an activator of the Wnt/β-catenin pathway and a stabilizer of β-catenin in melanoma, the pathways by which PDPN modulates PD-L1 have not yet been fully delineated. The collective evidence from our work supports a mechanistic axis whereby β-catenin underlies immunosuppression in the TME through the induction of PD-L1 and reinforces the therapeutic rationale for co-targeting the PDPN-β-catenin-PD-L1 pathway to enhance responses to immune checkpoint blockade. Additionally, our findings suggest that PDPN could serve as a predictive biomarker for immunotherapy resistance in cancer patients.

## Materials and methods

### Cell culture and cell transfection

A375, B16-F10, and HEK293T cells were maintained in DMEM (Gibco, South America) supplemented with 10% fetal bovine serum (FBS) and 1% penicillin-streptomycin (Beyotime, Shanghai), and cultured at 37 °C in a humidified atmosphere containing 5% CO_2_. A375 and B16-F10 cells were obtained from the Army Medical University (Chongqing, China), while the HEK293T line was sourced from ATCC. PDPN-targeted shRNA plasmids, cDNA fragments for PDPN and negative control particles were purchased from Shangwei Biotechnology (Shenzhen, China). Lentiviral packaging was conducted in HEK293T cells using UltraFection 3.0 transfection reagent (4A Biotech, Beijing), following the manufacturer’s protocol. Harvested lentiviral particles were transduced into A375 and B16-F10 cells in the presence of 10 μg/mL polybrene (Sigma), and transduced cells were selected with 5 μg/mL puromycin (Sigma). For PDPN overexpression, pcDNA3.1-CTNNB1 (human/mouse) or empty vector was stably integrated into A375 and B16-F10 cells under 5 μg/mL puromycin selection. All PDPN knockdown and CTNNB1 overexpression events were verified by western blotting.

### Western blotting assay

A375, B16-F10, A375 scramble, A375 PDPN shRNA, B16-F10 scramble, and B16-F10 PDPN shRNA cells were plated into 60-mm dishes. Cells were left untreated or subjected to CY12-RP2 (Qiang Yao, Shanghai) at indicated concentrations (20 µM) for 24 hours. Following treatment, cells were lysed in ice-cold RIPA buffer (MCE, Cat# HY-K1001) with protease inhibitor cocktail (MCE, Cat# HY-K0010). Proteins were separated by SDS-PAGE and transferred to PVDF membranes (Thermo Fisher Scientific). Blots were incubated with primary antibodies (see **Supplementary Table 4**) and HRP-conjugated, species-matched secondary antibodies. Bands were visualized using an ECL substrate (4A Biotech, Beijing, China) and imaged with a ChemiDoc instrument (Bio-Rad). Densitometric quantification was performed in ImageJ, and protein levels were normalized to β-actin as loading controls.

### Flow cytometry analysis

Membrane-associated PD-L1 expression was quantified by flow cytometry using a fluorochrome-conjugated anti-PD-L1 antibody. A375, B16-F10, A375 scramble, A375 PDPN shRNA, B16-F10 scramble, and B16-F10 PDPN shRNA cells were seeded into 60-mm dishes and treated with CY12-RP2 at specified concentrations (20 µM) or left untreated. After 48 hours, cells were collected, washed twice in ice-cold PBS, and incubated with APC anti-human/mouse PD-L1 (393610, 124312; Biolegend) for 30 min at 4°C to minimize internalization. After two further PBS washes, cells were resuspended in PBS for immediate flow cytometry analysis. Acquisition was performed using a CytoFLEX cytometer (Beckman Coulter, Brea, CA) and data analyzed in FlowJo (v10.7.1; Tree Star, Ashland, OR).

### Immunofluorescence analysis

A375, B16-F10, A375 scramble, A375 PDPN shRNA, B16-F10 scramble, and B16-F10 PDPN shRNA cells were grown on confocal-grade dishes (NEST) and left untreated or exposed to CY12-RP2 at designated concentrations (20 µM). After 24 hours, cells were fixed in 4% paraformaldehyde (PFA, 15 min), blocked with PBS + 1% BSA for 30 min at room temperature, then incubated overnight at 4°C with anti-PD-L1 primary antibody (1:500, Cat##86744, CST) in blocking buffer. After three 5-min washes in 0.01% Triton X-100/PBS, cells were incubated with ABflo^®^ 488-conjugated secondary antibody (1:1000, Goat anti-Mouse, Abclonal, Cat# AS037) for 1 h at room temperature and counterstained with DAPI for 5 min. Following final washes, cells were mounted in PBS:glycerol (1:1). Imaging was performed using a Leica TCS SP8 confocal microscope (63×/1.4 NA oil objective). Maximum intensity projections and image analyses were conducted in Fiji/ImageJ (NIH).

### Multiplex immunofluorescence in human melanoma tissue microarrays and mouse tissues

Multiplex immunofluorescence staining was performed on a human melanoma tissue microarray (Cohort ID: HMelC112CD01) containing 112 cores—benign nevi (n=1), primary cutaneous melanomas (n=94), and distant metastases (n=17)—to assess the associations between PDPN expression, immune infiltration, and PD-L1 levels. FFPE sections underwent antigen retrieval (10 mM citrate buffer, pH 6.0, 95°C, 30 min), then simultaneous incubation with primary antibodies against PDPN (1:200, 11629-1-AP, Proteintech), CD8 (1:200, 66868-1-Ig, Proteintech), and PD-L1 (1:200, 28076-1-AP, Proteintech) for 2 hours at room temperature, followed by incubation with ABflo^®^ 488-, 594-, or 647-conjugated goat anti-rabbit IgG (1:500 each, AS037, AS039, AS060) in PBS/1% BSA/1% goat serum (30 min, RT), all performed under coverslips with three 5-min PBS washes after each step. Nuclei were stained with DAPI (0.1 µg/mL, 5 min), and slides mounted with ProLong Diamond Antifade. Quantitative analysis of tissue microarray immunofluorescence was performed using the AQUA technique of QIF (NavigateBP). This method quantifies the target signal by calculating the quotient of the total target pixel intensity over the area of the molecularly designated compartment.

For parallel assessment of CY12-RP2-mediated immune modulation in C57BL/6 melanoma models, lymph nodes, spleens, and tumors were harvested, sectioned into sequential 12 µm cryosections, fixed in ice-cold acetone, blocked with 5% goat serum (1 h), and incubated with CD3 (1:200, 17617-1-AP, Proteintech), CD8 (1:200, 29896-1-AP, Proteintech), and Granzyme B (1:200, 13588-1-AP, Proteintech) antibodies for 2 hours at room temperature, followed by multiplex ABflo^®^ secondary antibody staining using identical protocols as above. Imaging was performed using a Nikon confocal microscope, and cell quantification was carried out in QuPath v0.4.3 using tissue segmentation (DAPI threshold), cellular phenotyping (≥50 cells/mm² threshold), and spatial proximity analysis (≤15 µm between cells).

### Luciferase reporter assay

A375 melanoma cells were maintained in DMEM supplemented with 10% FBS and 1% penicillin-streptomycin at 37°C with 5% CO_2_. For luciferase assays, A375 cells seeded in 24-well plates were co-transfected at 70–80% confluency using Lipofectamine 3000. Each well received the pGL3-CD274 promoter construct (0.5 μg), pRL-TK (0.05 μg), and increasing amounts of β-catenin overexpression plasmid (0–0.5 μg; empty vector balanced total DNA) (Shangwei Biotechnology, Shenzhen). After 48 hours, cells were lysed, and luciferase activity was measured using the Dual-Luciferase Reporter Assay (11402ES60, YEASEN). Firefly luciferase readings were normalized to Renilla to determine relative promoter activity.

### Enzyme-linked immunosorbent assay

The levels of major inflammatory cytokines, including TNF-α (BMS607-3TEN, Invitrogen), IL-1β (BMS6002, Invitrogen), IL-2 (BMS601, Invitrogen), and IFN-γ (BMS606-2, Invitrogen) were measured using an ELISA kit. Briefly, serum of C57BL/6 melanoma models was collected and added to an antibody pre-coated 96-well plate, and then followed up according to the manufacturer’s instructions.

### RNA-seq analysis

RNA-seq libraries were generated from 1 μg total RNA per sample, using the Illumina TruSeq Stranded mRNA Library Prep Kit per manufacturer instructions. Paired-end sequencing (150 bp reads) was performed on an Illumina NovaSeq 6000. Data analysis proceeded via established protocols with specified modifications: Raw quality was assessed with FastQC (v0.10.0); adapter trimming and quality filtering (Phred score &gt;20) were performed using TrimGalore! (v0.6.4). Processed reads were mapped to the GRCm39 GENCODE vM30 reference using STAR (v2.7.3a). Gene expression counts were obtained with HTSeq-count (v0.11.2), and differential expression was determined by DESeq2 (v1.32.0) with cutoffs of |log_2_FC| &gt; 0.5 and FDR-adjusted p&nbsp;&lt;&nbsp;0.05. All computations were conducted in R (v4.1.0). Genes with FPKM ≥1 in at least 3 samples were subjected to KEGG enrichment analysis using DAVID (v6.8).

### *In vivo* tumor growth assays

All animal experiments were approved by the Jiangxi University of Chinese Medicine Animal Ethics Committee (Protocol NYLLSC 20250415) and performed in accordance with China’s Guidelines for Ethical Review of Laboratory Animal Welfare (GB/T 35892-2018). For tumorigenicity assays, 4–6-week-old female BALB/c nude mice and C57BL/6 mice (Guangdong Medical Laboratory Animal Center) were injected subcutaneously in the flank with 1×10^6^ B16-F10 cells in 100 μL PBS. Four days post-implantation, animals were randomized into control and treatment groups (n=5) using block randomization. From the sixth day, animals received intravenous injections of CY12-RP2 (50 mg/kg) or vehicle every 48 hours. Tumor volumes (calculated as 0.5 × L × W²) and body weights were measured three times weekly over 21 days. At study endpoint, tumors were excised, photographed, and fixed in 4% paraformaldehyde for 24 hours; spleens and lymph nodes underwent identical fixation for H&E histopathology.

For CD8^+^ T cell depletion experiments, C57BL/6 mice bearing B16-F10 tumors received intraperitoneal administration of anti-CD8α antibody (200 μg; BE0061, BioXCell) on days 1, 4, and 9 following tumor inoculation (day 0). On day 4, animals were randomized to receive CY12-RP2 (50 mg/kg) every 48 hours. Tumor volumes and body weights were tracked throughout the 18-day experiment. At endpoint, tumors were excised, documented, and fixed in 4% paraformaldehyde.

### TUNEL assay

TUNEL assay was conducted to evaluate apoptosis in paraffin-embedded tumor tissue sections with a cell death detection kit (MCE, HY-K1078). Following deparaffinization and rehydration, sections were subjected to antigen retrieval in sodium citrate buffer (pH 6.0) at 60 °C for 4 hours, and then permeabilized with 0.3% Triton X-100 (in PBS, pH 7.4) for 15–30 min. Each tissue section was covered with 50 µL of TUNEL working solution, and incubated at 37°C for 1 hour. After PBS washes the next day, nuclei were stained with DAPI. Imaging was performed using a fluorescence microscope (Nikon, Japan).

### Quantification and statistical analysis

All quantitative analyses were performed using GraphPad Prism 9 and results are presented as mean ± SD. Cell-based measurements, including migration distance and proliferation counts, were quantified using ImageJ-based morphometric analysis. Statistical comparisons between groups used unpaired, two-tailed Student’s t-tests for normally distributed data, with significance thresholds: *p < 0.05, **p < 0.01, ***p < 0.001, ****p < 0.0001; ns. (not significant) = p ≥ 0.05.

## Results

### PDPN correlates with immunosuppressive landscapes in melanoma

**Figure 1:**
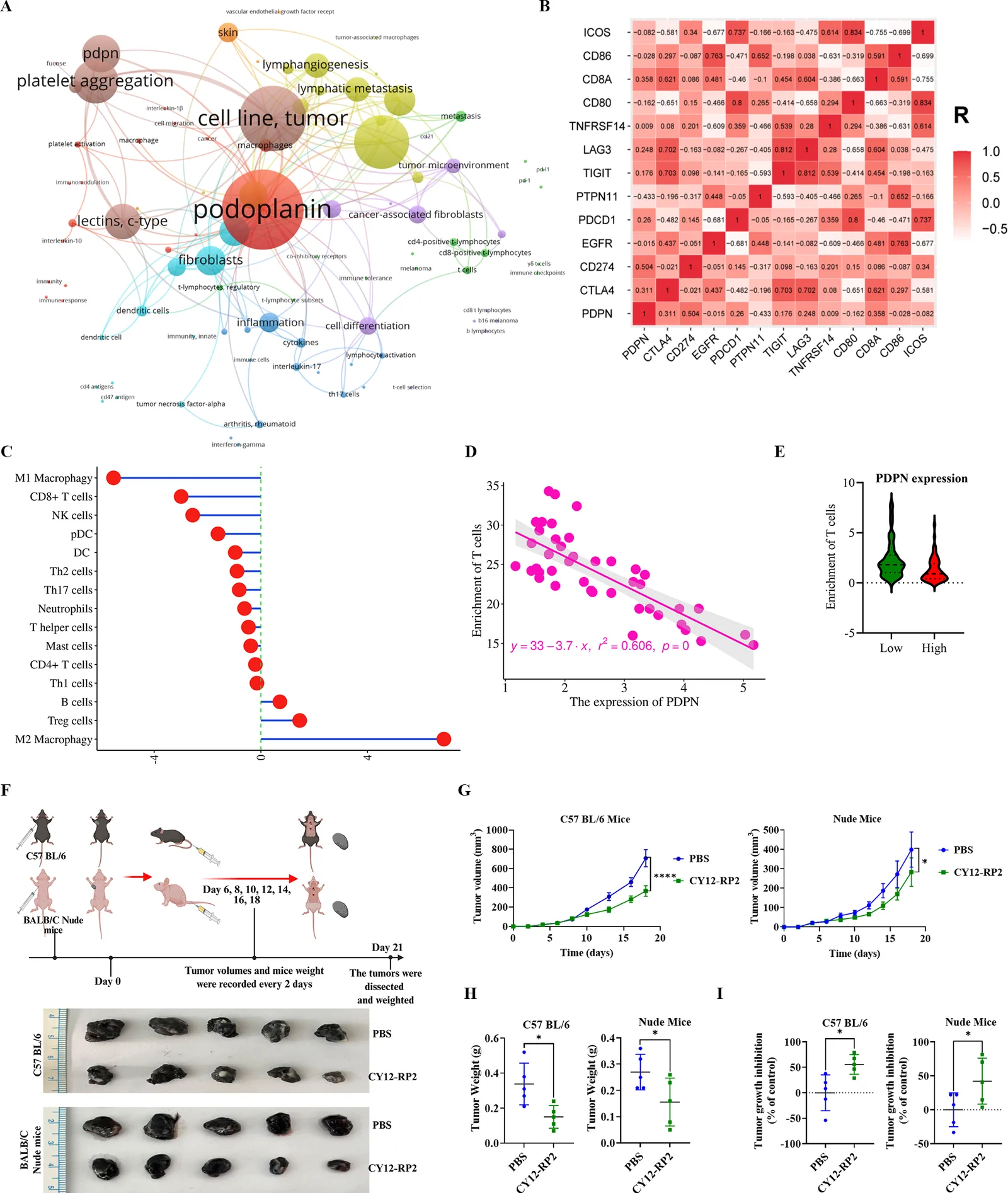
PDPN correlates with an immunosuppressive landscape in melanoma. **(A)** Keyword cluster analysis by VOSviewer. **(B)** Correlation of PDPN and immune checkpoint receptors using TCGA datasets (https://portal.gdc.cancer.gov/). **(C–E)** Single-cell RNA-seq analysis of 42 melanoma samples from TCGA. **(F)** B16-F10 cells (1×10^6^) were injected subcutaneously into both BALB/c nude and C57BL/6 mice. Starting on day 6, mice received CY12-RP2 (50 mg/kg) or vehicle every two days (n = 5 per group). Tumor size and body weight were measured every two days. **(G)** Growth curves of subcutaneous xenografts in both mouse models. **(H)** Terminal tumor weights. **(I)** Tumor inhibition rates per strain. Data represent mean ± SD. *p < 0.05, ****p < 0.0001 vs. PBS.

PDPN functions as a pivotal orchestrator of immune checkpoint networks and contributes to the establishment of immunosuppressive landscapes (5, 8). Cluster analysis using VOSviewer identified PDPN-associated modules encompassing platelet aggregation, tumor microenvironment, inflammation, lymphatic metastasis, and CD8^+^ T lymphocyte biology (**Figure 1A**). Evaluation of melanoma data from The Cancer Genome Atlas (TCGA) revealed strong positive correlations between PDPN expression and several immune checkpoint receptors, including PD-L1, CTLA4, LAG3, TIGIT, and BTLA, with the association with PD-L1 (CD274) being the most prominent (r=0.504, p<0.001; **Figure 1B**). STRING database analysis further substantiated the direct physical and functional interactions between PDPN and these immune checkpoints. Additionally, PDPN demonstrated significant network connectivity (FDR<0.05) with major immune lineage markers (CD86, CD8A, CD4, CD80; **Supplementary Figure S1B**). TIMER2.0 analysis of 471 melanoma samples (368 metastatic, 103 primary) by single-cell RNA-seq revealed that PDPN expression was inversely correlated with infiltration by antitumor immune populations (CD4^+^ T cells, CD8^+^ T cells, NK cells, and M1 macrophages), and positively correlated with immunosuppressive populations (M2 macrophages, regulatory T cells), most notably with M2 macrophages (**Supplementary Table 1**). These associations were independently validated using a separate single-cell RNA-seq TCGA cohort (n=42), which demonstrated that PDPN modulates immune cell infiltration, suppresses cytotoxic immune cell functions, and inversely correlates with T cell accumulation within the tumor (**Figures 1C–E**).

TTo directly evaluate the immunomodulatory role of PDPN, the inhibitory peptide CY12-RP2 was tested in melanoma xenograft models using both immunodeficient BALB/c nude mice and immunocompetent C57BL/6 mice. As shown in **Figure 1G**, administration of CY12-RP2 led to a significant reduction in tumor growth rates relative to controls in both animal models. CY12-RP2-treated cohorts showed substantial decreases in both tumor volume and terminal tumor weight (**Figures 1F, H**), achieving tumor inhibition rates of 42.22% in BALB/c nude mice and 60.62% in C57BL/6 mice (**Figure 1I**). These data indicate that CY12-RP2 exerts enhanced antitumor effects in immunocompetent hosts compared to immunodeficient counterparts.

### PDPN promotes an immunosuppressive microenvironment in melanoma through PD-L1 co-expression and reduction of CD8^+^ T cells

**Figure 2:**
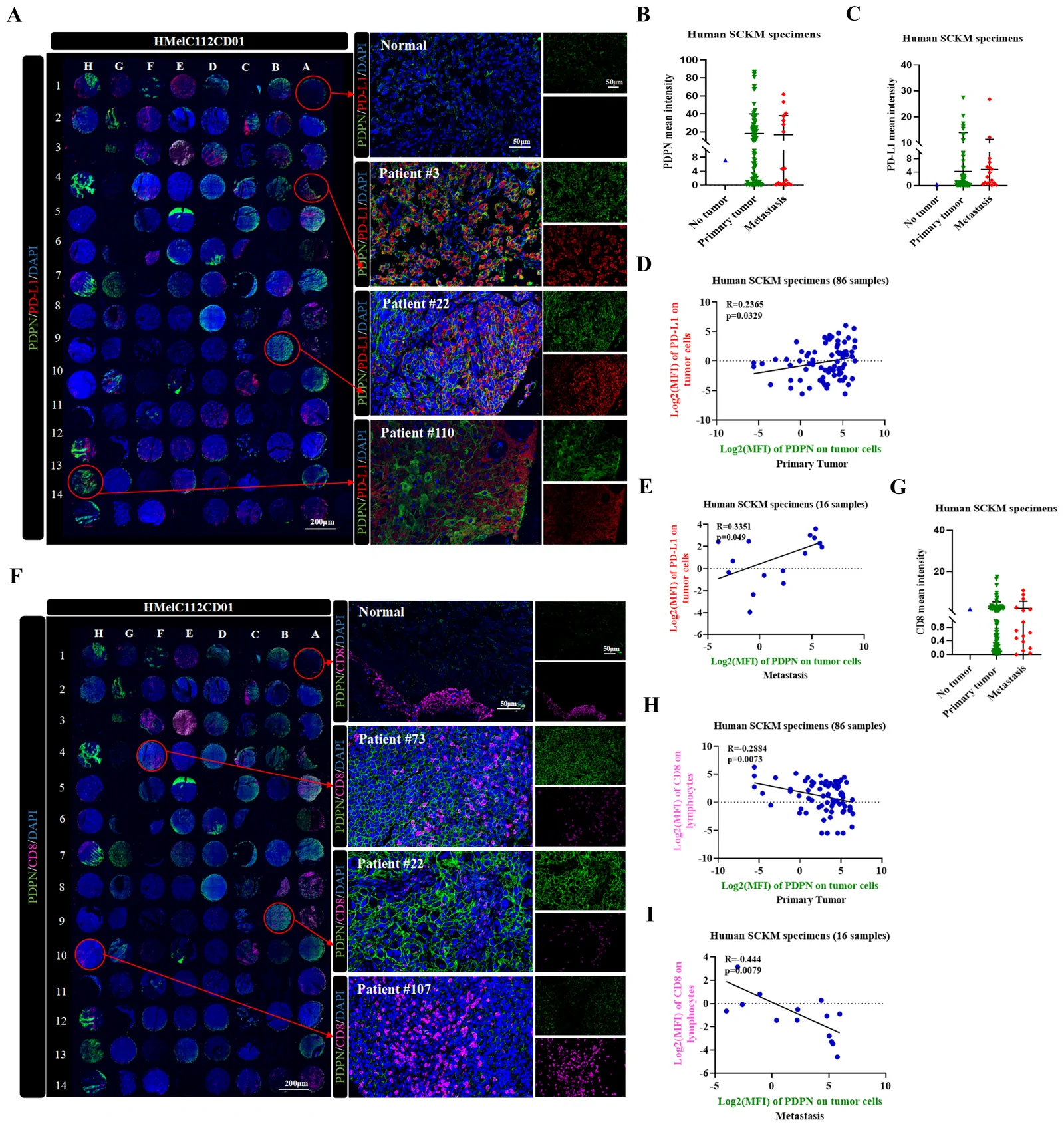
PDPN promotes an immunosuppressive microenvironment in melanoma through PD-L1 co-expression and reduction of CD8^+^ T cells. **(A)** Representative multiplex immunofluorescence images of PDPN and PD-L1 in melanoma TMA. Scale bars: 200 µm and 50 µm. **(B, C)** Quantitative expression analysis of PDPN and PD-L1 in non-tumor tissues, primary melanoma, and metastases. **(D, E)** Correlation analysis of PDPN and PD-L1 co-expression in primary and metastatic melanoma samples. **(F)** Multiplex immunofluorescence images of PDPN and CD8 in TMA. Scale bars: 200 µm and 50 µm. **(G)** Quantitative analysis of CD8^+^ T cells infiltration. **(H, I)**. Correlation between PDPN expression and CD8^+^ T cells infiltration in primary and metastatic specimens.

Integrated multi-database analysis revealed a significant positive association between PDPN and the immunosuppressive checkpoint receptor PD-L1 in melanoma transcriptomes. To confirm this, a human tissue microarray (HMelC112CD01, Shanghai Xinchao Biotechnology) comprising 112 melanoma specimens was assessed (19). Triple immunofluorescence staining showed limited co-expression of PDPN and PD-L1 in benign nevi, whereas marked upregulation and co-expression were observed in primary (e.g., #3, #22) and metastatic (e.g., #110) melanoma lesions (**Figure 2A**). Semi-quantitative assessment of 111 melanoma specimens revealed a correlation between PDPN and PD-L1 expression (overall co-expression 46.1%, p < 0.05), with disease stage-specific disparities: PDPN prevalence was higher in primary lesions (61.6%) than in metastases (43.8%), whereas PD-L1 expression was elevated in metastatic lesions (87.5%) compared to primary tumors (61.6%). Notably, the co-expression rates of PDPN and PD-L1 were consistent across disease stages (46.5% in primary versus 43.8% in metastatic lesions), corroborated by transcriptomic analyses (**Figures 2B–E**, **Supplementary Table 2**). Spatial profiling using multiplex immunofluorescence for PDPN and CD8 on tissue microarrays revealed distinct mutual exclusion: regions with high PDPN expression exhibited sparse CD8^+^ T cell infiltration (e.g., #22, #73), while PDPN-low/negative regions corresponded to dense CD8^+^ T cell accumulation (e.g., #107) (**Figures 2F, G**). Specifically, PDPN-high areas experienced a 4.2-fold reduction in CD8^+^ T cell density versus PDPN-low counterparts (p < 0.001), indicating a central role for PDPN in T cell exclusion (**Supplementary Table 3**). This inverse correlation was validated in both primary (r = −0.2884, p < 0.05; **Figure 2H**) and metastatic (r = −0.444, p < 0.01; **Figure 2I**) melanomas, substantiating PDPN’s dual immunosuppressive role in immune checkpoint activation and impairment of T cell infiltration, in agreement with transcriptomic deconvolution data.

Collectively, these results suggest that PDPN may function as a potential therapeutic hub, coordinating the activation of immunosuppressive checkpoints and the exclusion of T cells in melanoma. This proposed mechanistic role is supported by consistent associations between PDPN and multiple immune checkpoint markers across independent transcriptomic datasets, including the TISIDB (**Supplementary Figure S1A**) and TCGA pan-cancer cohorts (**Figure 1B**).

### PDPN regulates cell surface PD-L1 expression in melanoma cells

**Figure 3:**
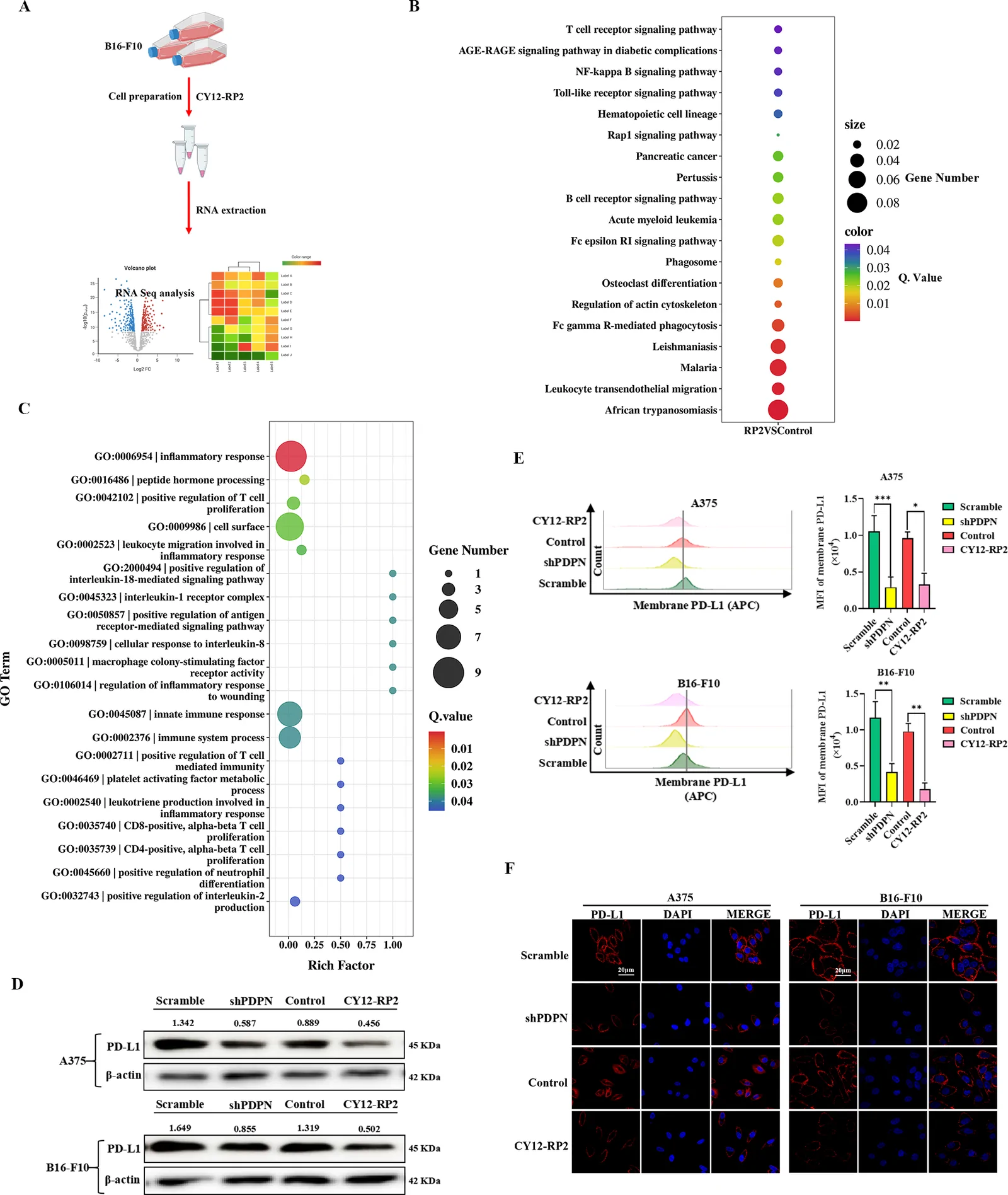
PDPN regulates cell surface PD-L1 expression in melanoma cells. **(A)** RNA-seq profiling workflow: RNA-seq was performed on B16-F10 cells treated with CY12-RP2 and untreated controls. **(B)** KEGG pathway enrichment analysis: Significantly enriched pathways (p < 0.05) are shown. Dot size corresponds to the number of genes mapped to each pathway, color scale corresponds to the adjusted Q-value of the enriched terms. **(C)** Gene Ontology (GO) enrichment analysis: Terms with a Q value ≤ 0.05 were considered significantly enriched. **(D)** Western blot analysis of PD-L1 expression in PDPN-knockdown and CY12-RP2-treated (20 µM) A375 and B16-F10 cells. **(E)** Flow cytometry-based measurement of membrane PD-L1 expression in PDPN-knockdown and CY12-RP2-treated A375 and B16-F10 cells. Statistical comparisons are between the indicated samples and the control. **(F)** Immunofluorescence staining of membrane PD-L1 in PDPN-knockdown and CY12-RP2-treated A375 and B16-F10 cells. Scale bar: 20 μm. Data represent mean ± SD. *p < 0.05, **p < 0.01, ***p < 0.001 vs. Control/Scramble.

RNA-seq analysis of CY12-RP2–treated B16-F10 melanoma cells identified differential expression of 1,228 genes, including 572 upregulated and 656 downregulated genes, relative to controls (**Figure 3A**). KEGG enrichment analysis demonstrated significant activation (p < 0.05) of immune-related pathways, including T cell receptor, NF-κB, Toll-like receptor, and FcγR-mediated phagocytosis signaling, following CY12-RP2 treatment (**Figure 3B**), revealing its broad immunostimulatory properties. Gene Ontology analysis revealed enhanced regulation of T cell proliferation, inflammatory response, and immune system processes (**Figure 3C**). Parallel transcriptional alterations between CY12-RP2 exposure and genetic PDPN knockdown confirm the PDPN-dependent nature of this immune reprogramming. Importantly, PDPN inhibition counteracts immune escape in melanoma through the downregulation of immune checkpoint receptors (PD-1, CTLA-4) and reconstitution of antitumor immunity, emphasizing PDPN’s role as a key immunosuppressive regulator and highlighting CY12-RP2 as a promising candidate for immunotherapy.

To dissect the mechanistic basis for PDPN-driven immunosuppression, PD-L1 expression was assessed in melanoma cells following either PDPN knockdown or CY12-RP2 treatment. Western blot analysis demonstrated a marked reduction in total PD-L1 protein levels in A375 and B16-F10 melanoma cells upon PDPN depletion (**Figure 3D**). Likewise, CY12-RP2 treatment significantly reduced PD-L1 protein expression relative to controls (**Figure 3D**). Recognizing that cell surface-localized PD-L1 on tumor cells engages PD-1 on T cells to inhibit antitumor responses, we further quantified cell surface PD-L1 using flow cytometry and immunofluorescence in A375 and B16-F10 melanoma cells. Both PDPN knockdown and CY12-RP2 treatment significantly decreased PD-L1 cell surface levels (**Figures 3E, F**), confirming that PDPN regulates the biologically active, cell surface population of PD-L1. Taken together, these findings establish that PDPN controls the expression of cell surface PD-L1 in melanoma cells.

### PDPN promotes PD-L1 mRNA transcription in melanoma through activation of a β-catenin-dependent mechanism

**Figure 4:**
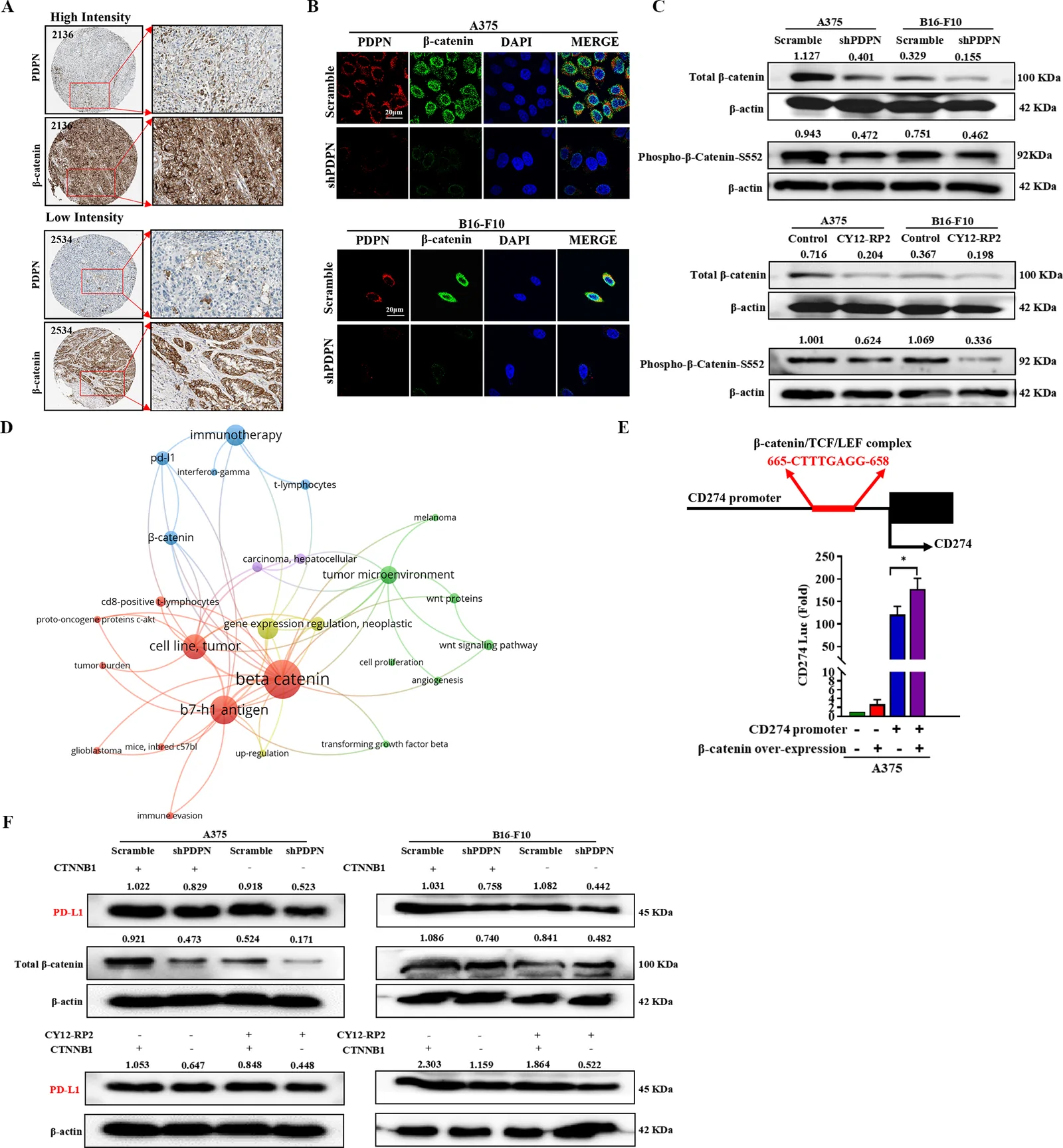
PDPN promotes PD-L1 mRNA transcription in melanoma through activation of a β-catenin-dependent mechanism. **(A)** The protein levels of PDPN and β-catenin in in human melanoma specimens from the Human Protein Atlas database, as detected by IHC staining. **(B)** Immunofluorescence staining of PDPN and β-catenin in PDPN-knockdown A375 and B16-F10 cells. Scale bar: 20 μm. **(C)** Western blot analysis of phospho-β-catenin (S552) and total β-catenin expression in PDPN-knockdown and CY12-RP2-treated (20 µM) A375 and B16-F10 cells. β-actin was used as a loading control. **(D)** Keyword cluster analysis by VOSviewer. **(E)** The putative β-catenin/TCF/LEF binding site on CD274 promoter sequence determined by online PROMO algorithm. The luciferase activity of A375 cells by luciferase reporter assays. **(F)** Western blot analysis of PD-L1 and total β-catenin expression in PDPN-knockdown and CY12-RP2-treated (20 µM) A375 and B16-F10 cells upon CTNNB1 overexpression. β-actin served as a loading control. Data represent mean ± SD. *p < 0.05, vs. control.

To clarify the molecular mechanism underlying PDPN-mediated regulation of PD-L1, we examined whether β-catenin signaling is involved in this regulatory axis. Our previous study has shown that PDPN hyperactivates the Wnt/β-catenin pathway in melanoma by stabilizing β-catenin, promoting its nuclear translocation and complex formation with LEF/TCF, and driving oncogenic transcriptional programs (19). Analysis of the Human Protein Atlas (HPA, https://www.proteinatlas.org/) revealed a strong positive correlation between PDPN and β-catenin protein expression in human melanoma specimens (**Figure 4A**). Immunofluorescence analysis further revealed that β-catenin expression is significantly reduced in A375 and B16-F10 melanoma cells following PDPN knockdown, and that both total and S552-phosphorylated β-catenin levels are significantly decreased upon PDPN depletion or CY12-RP2 treatment (**Figures 4B, C**). As Wnt/β-catenin signaling is a primary modulator of PD-L1 expression, co-occurrence analysis using VOSviewer highlighted a strong association between PD-L1 and β-catenin (**Figure 4D**). The online PROMO tool was used to identify presumptive β-catenin/TCF/LEF binding sites in the CD274 promoter. Luciferase reporter assays demonstrated that co-transfection of β-catenin overexpression constructs and CD274-luciferase vectors into A375 cells significantly increased reporter activity compared to controls (**Figure 4E**). Notably, overexpression of CTNNB1 (β-catenin) fully restored PD-L1 levels following PDPN knockdown or CY12-RP2 treatment, as shown by immunoblotting (**Figure 4F**). These findings collectively establish β-catenin as an essential downstream mediator of PDPN-driven PD-L1 expression in melanoma.

Together, our integrated transcriptomic and proteomic analyses support a mechanistic model in which PDPN activates Wnt/β-catenin signaling, leading to transcriptional upregulation of PD-L1 and contributing to the formation of an immunosuppressive microenvironment in melanoma.

### The inhibition of B16-F10 tumor growth by CY12-RP2 is CD8+ T cell-dependent

**Figure 5:**
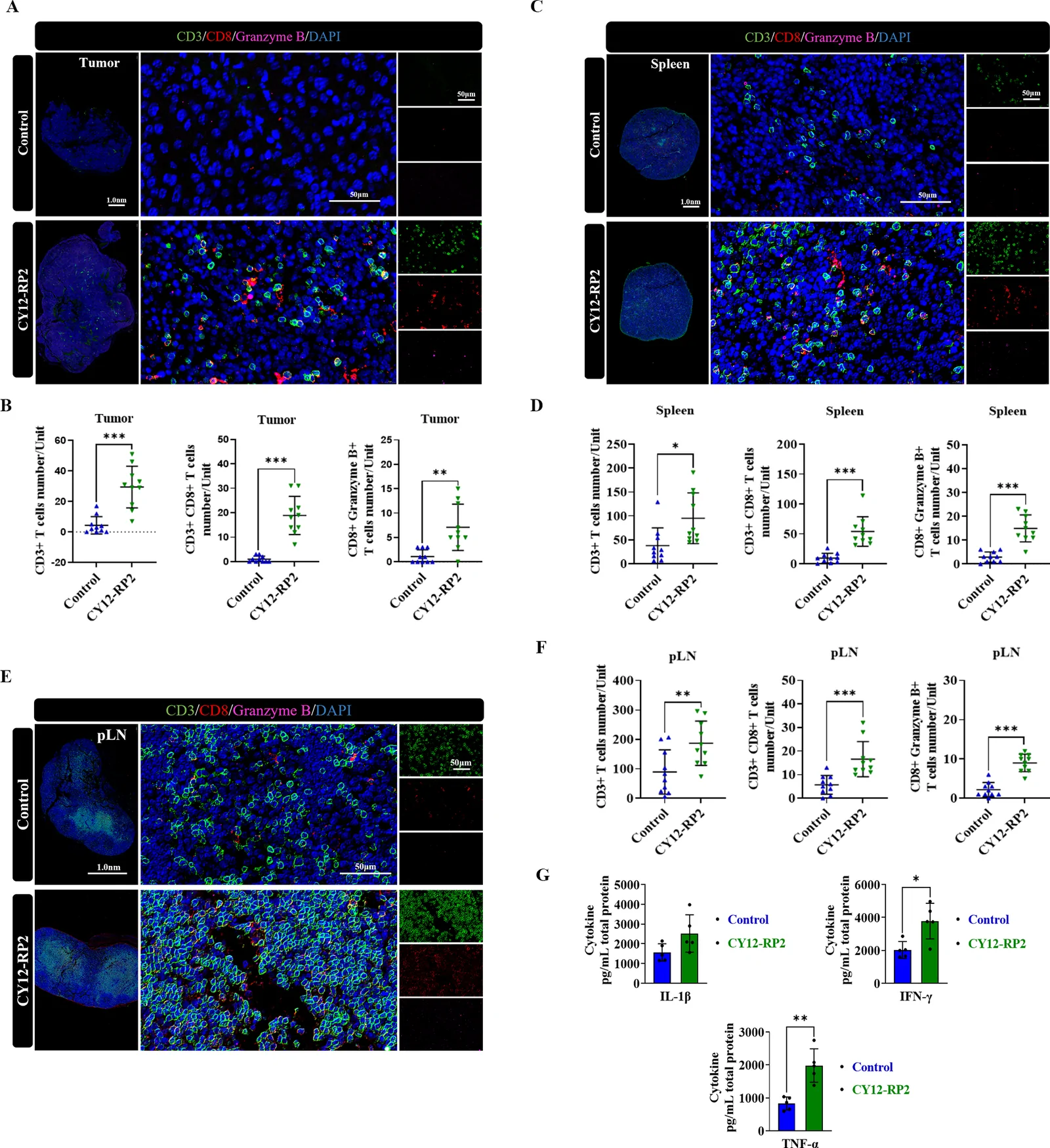
The inhibition of B16-F10 tumor growth by CY12-RP2 is CD8+ T cell-dependent. Multiplex immunofluorescence staining was employed to evaluate proportional changes and tissue infiltration of CD3^+^ T cells, CD8^+^ T cells, and Granzyme B^+^ cells within tumor tissues, spleens, and lymph nodes in treated and control groups. **(A)** Representative multiplex immunofluorescence images of tumor tissues. Scale bars: 1.0 nm and 50 µm. **(B)** Quantification of immune cell infiltration within tumors. **(C)** Representative spleen images. Scale bars: 1.0 nm and 50 µm. **(D)** Analysis of immune cell proportions in spleens. **(E)** Representative lymph node images. Scale bars: 1.0 nm and 50 µm. **(F)** Assessment of immune cell proportions in lymph nodes. **(G)** Serum cytokine concentrations (IFN-γ, TNF-α, IL-1β) in C57BL/6 mice as measured by ELISA. Data are expressed as mean ± SEM. *P < 0.05, **P < 0.01, ***P < 0.001.

To delineate the contribution of lymphocytes to CY12-RP2-mediated suppression of B16-F10 melanoma growth, multiplex immunofluorescence was used to assess immune cell infiltration in tumors, spleens, and lymph nodes from syngeneic tumor-bearing mice. CY12-RP2 treatment significantly increased the density of tumor-infiltrating lymphocytes (p < 0.001), as evidenced by expansions in CD3^+^ T cells and prominent elevations in Granzyme B^+^CD8^+^ cytotoxic T lymphocytes (72.4% in CY12-RP2–treated mice vs 41.8% in controls; p < 0.01; **Figures 5A, B**). Immune profiling of peripheral tissues identified elevated frequencies of cytotoxic CD8^+^ T cells in the spleens and lymph nodes of CY12-RP2–treated tumor-bearing mice (**Figures 5C–F**), indicating that CY12-RP2 promotes systemic immune activation. CY12-RP2 also remodeled the systemic immunity cytokine milieu, increasing key pro-inflammatory mediators such as IFN-γ, TNF-α, and IL-1β. These cytokine levels were significantly higher in serum from CY12-RP2–treated mice (**Figure 5G**), suggesting a shift toward a pro-inflammatory, antitumor immune contexture in the systemic immunity. IFN-γ, which sensitizes tumor cells to T cell-mediated cytotoxicity, and other cytokines remained substantially elevated in serum. Collectively, these results demonstrate that CY12-RP2 functions as a multifaceted immune modulator, counteracting tumor-induced immunosuppression by both expanding cytotoxic lymphocyte populations across lymphoid compartments and reprogramming the tumor microenvironment to support immune-mediated tumor clearance.

### Depletion of CD8^+^ T cells abrogates the *in vivo* antitumor efficacy of CY12-RP2

**Figure 6:**
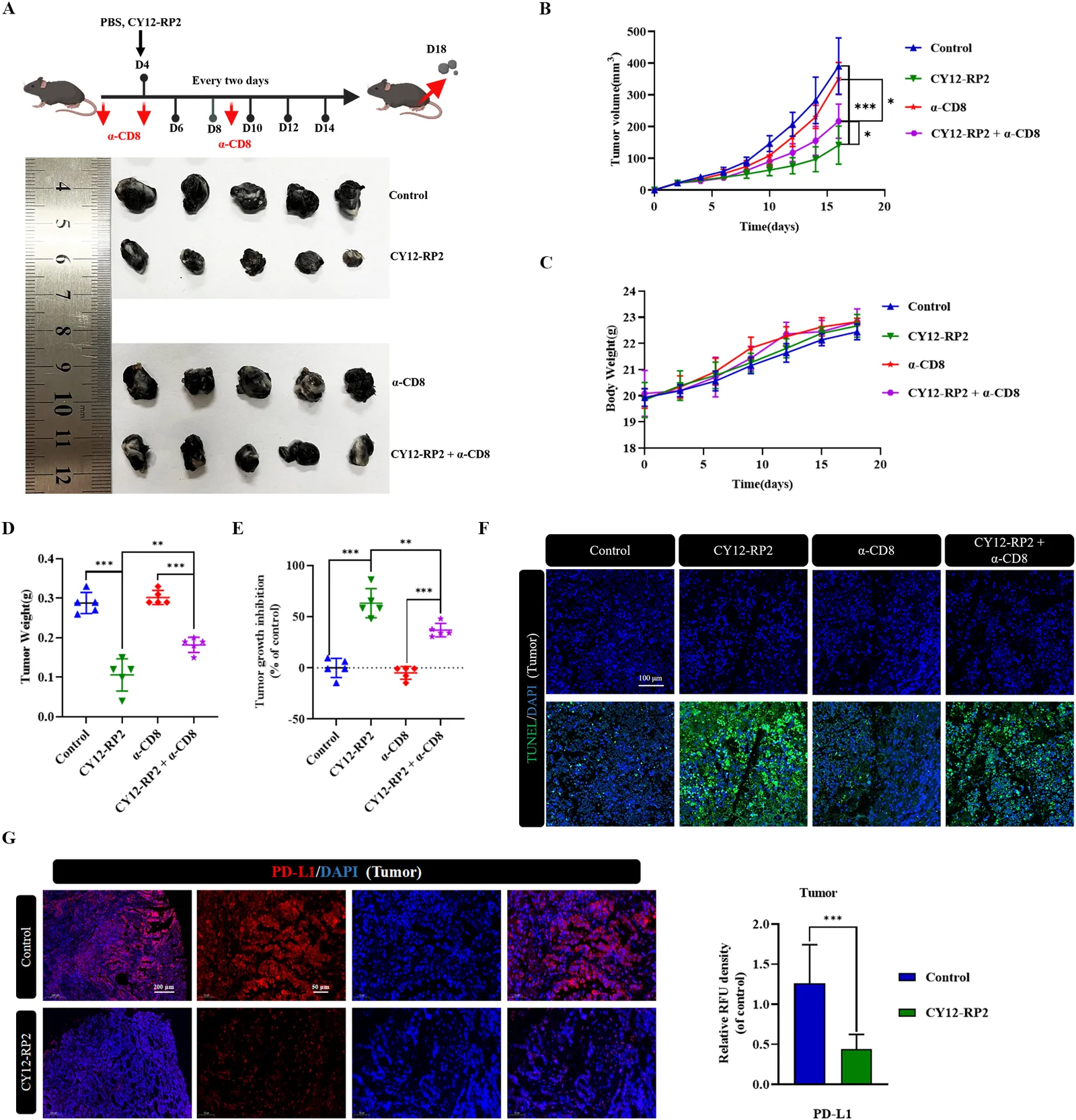
Depletion of CD8^+^ T cells abrogates the *in vivo* antitumor efficacy of CY12-RP2. **(A)** C57BL/6 mice with B16-F10 tumors were administered anti-CD8α antibodies intraperitoneally on days 1, 4, and 9 post-inoculation (day 0). By day 4, animals were randomized to receive CY12-RP2 (50 mg/kg) or vehicle every 48 hours. Tumor volume and body weight were recorded throughout the 18-day period. **(B)** Tumor growth curves in C57BL/6 mice. **(C)** Changes in body weights among tumor-bearing mice. **(D)** Terminal tumor weights. **(E)** Tumor inhibition rates among groups. **(F)** Immunofluorescence staining of TUNEL in C57BL/6 mice melanoma tumors. Scale bar: 100 μm. **(G)** Immunofluorescence staining of PD-L1 in C57BL/6 mice melanoma tumors. Scale bar: 200 μm, 50 μm. Quantification of PD-L1 expression levels in melanoma tumors. Data represent mean ± SD. *p < 0.05, **p < 0.01, ***p < 0.001, vs. control group.

To directly test whether the antitumor effect of CY12-RP2 depends on CD8^+^ T cells, given their redistribution across tumor, spleen, and lymph node compartments, targeted depletion of CD8^+^ T cells was implemented using anti-CD8α antibodies in B16-F10 tumor-bearing C57BL/6 mice. Combination therapy with CY12-RP2 and anti-CD8α antibodies dramatically diminished tumor suppression compared to CY12-RP2 alone (**Figures 6A, B**). Importantly, there was no signifcant change in the mean body weight of C57BL/6 mice (**Figure 6C**). Quantitative assessment revealed that CD8^+^ T cell depletion reduced CY12-RP2 efficacy by 41.8% and 25.8% in two independent tumor models, with tumor inhibition declining from 63.2% to 36.8% and from 80.5% to 59.7%, respectively (both p < 0.001) (**Figures 6D, E**). These findings unambiguously demonstrate that the antitumor activity of CY12-RP2 is contingent upon intact CD8^+^ T cell effector functions. The attenuation of therapeutic benefit persisted despite ongoing CY12-RP2 administration during intermittent anti-CD8 treatment, with tumor volumes and body weight (with <5% variance) monitored throughout the experiment. Lack of Granzyme B^+^ CD8^+^ T cell infiltration in tumors from anti-CD8 antibody-treated mice further confirmed this mechanistic requirement. To evaluate CY12-RP2 efficacy, TUNEL assays were performed on tumors collected at day 18, revealing a significant increase in TUNEL^+^ apoptotic cells within the CY12-RP2 treatment group relative to controls. Importantly, co-treatment with anti-CD8 antibody markedly reduced the cytotoxic effects of CY12-RP2, confirming the essential role of CD8^+^ T cells in mediating its antitumor response (**Figure 6F**).

Recognizing the established link between cytokine signaling and PD-L1 induction, PD-L1 levels were assessed in tumors from CY12-RP2–treated mice. Immunofluorescence consistently showed substantial reductions in PD-L1 expression in tumor sections (as determined by immunofluorescence) and at the cell membrane (**Figure 6G**), supporting the notion that CY12-RP2 effectively targets and suppresses this crucial immunosuppressive pathway.

## Discussion

Melanoma is distinguished as a highly immunogenic cancer, with dense intratumoral lymphocytic infiltration frequently correlating with favorable clinical outcomes. While PDPN has attracted attention as a potential immunotherapeutic target, its specific mechanistic functions in melanoma, particularly regarding crosstalk with immune cells in the tumor microenvironment, remain inadequately clarified. Bioinformatic evidence demonstrates strong associations between PDPN expression and immune cell infiltration, immune landscape polarization, and upregulated PD-L1. Despite notable efficacy of PD-1/PD-L1 immune checkpoint blockade, overall patient responses remain limited, in part due to heterogeneity in PD-L1 expression. Therefore, defining the molecular basis of PD-L1 regulation is critically important, and PDPN’s involvement in this regulatory axis stands as a key unresolved issue in melanoma immunobiology.

Our tissue microarray analyses revealed a correlation between PDPN and PD-L1 expression in both primary (46.5% co-expression) and metastatic (43.8% co-expression) melanoma specimens. Functional studies demonstrated that PDPN fosters immune escape by promoting PD-L1 upregulation and impairing T cell–mediated antitumor responses. In immunocompetent mouse models, administration of the PDPN-targeting inhibitory peptide CY12-RP2 enhanced T cell infiltration and increased the cytotoxic activity of CD8^+^Granzyme B^+^ T cells, which are fundamental for perforin–granzyme B-dependent tumor cell lysis. CY12-RP2 additionally induced the production of pro-inflammatory cytokines, including IL-1β, TNF-α, and IFN-γ. Importantly, although IFN-γ and TNF-α can activate JAK–STAT1 (30, 31) and NF-κB/ERK1/2 (32) signaling pathways, promoting transcriptional upregulation of PD-L1, our data show that the inhibitory effect of CY12-RP2 on PD-L1 expression predominates, resulting in net PD-L1 downregulation. Mechanistically, PDPN knockdown or CY12-RP2 treatment suppressed Wnt/β-catenin signaling—an essential regulatory axis for PD-L1 expression. These results are consistent with reports from other cancer models: Du, Linyong et al. elucidated β-catenin as a direct transcriptional activator of PD-L1 in glioblastoma (26), while studies by Haihua Wang et al. and PARISA et al. have demonstrated β-catenin’s role in immune exclusion and PD-L1 upregulation in diverse tumors (33, 34). Our study confirms and expands upon these findings by establishing PDPN as an upstream regulator of β-catenin-dependent PD-L1 transcription in melanoma. Nevertheless, the mechanisms governing PDPN-mediated nuclear transport and transcriptional activity of β-catenin remain to be definitively resolved in future investigations.

Despite clear evidence implicating the PDPN–β-catenin–PD-L1 axis as a prominent immunosuppressive pathway, numerous aspects of PDPN-mediated immune regulation remain to be fully elucidated. While the present investigation highlights this regulatory axis, previous studies have reported additional signaling pathways involving PDPN. For example, Hwang et al. showed that the PDPN–CLEC-2 interaction on platelets not only facilitates tumor progression but also critically modulates immune responses. In Osteosarcoma models expressing PDPN, anti-CLEC-2 monoclonal antibody (2A2B10) treatment reduced plasma concentrations of key pro-inflammatory cytokines and suppressed lung metastasis relative to control mice (35). Inhibition of the PDPN–CLEC-2 axis thus alleviates local immunosuppression. Beyond effects on tumor cells, PDPN also contributes to immune escape via the stromal compartment. For instance, PDPN-expressing cancer-associated fibroblasts (PDPN^+^ CAFs) are associated with an immunosuppressive tumor microenvironment characterized by increased CD204+ tumor-associated macrophage infiltration, while CD8+ and FOXP3+ TILs did not. (16, 17). These Tregs are recognized to secrete immunosuppressive cytokines, such as IL-10, IFN-γ, and TGF-β, which undermine CD8^+^ T cell function and facilitate immune evasion (36, 37). PDPN^+^ CAFs thus represent both a biomarker and active mediator of TGF-β–driven immunosuppression and therapy resistance. Cell-intrinsically, PDPN can also regulate T cell function bidirectionally. In T cell-specific PDPN-deficient mice, loss of PDPN exacerbates autoimmunity, while overexpression inhibits T cell responses and mitigates neuroinflammation (38). PDPN on lymph node stromal cells has been shown to suppress T cell proliferation and enhance tumor growth by restricting antitumor CD4^+^ T cell function, whereas depletion of stromal PDPN bolsters antitumor T cell activity and limits tumor progression (39). Moreover, interactions between PDPN and CLEC-2 directly modulate dendritic cell (DC) biology by activating Rac1 and inhibiting RhoA GTPase, instigating cytoskeletal remodeling and facilitating DC migration, which is critical for mounting immune responses in immunologically privileged sites (40). Collectively, these data demonstrate that PDPN regulates immune cell function and cytokine production via varied signaling cascades beyond its influence on PD-L1, underscoring the multifaceted character of its immunomodulatory functions. The emerging evidence suggests that additional, as yet unidentified, mechanisms likely contribute to the broader spectrum of PDPN-mediated immune regulation.

This study is not without limitations. Although multiplex immunofluorescence revealed marked increases in antitumor CD8^+^ T cell infiltration upon CY12-RP2 treatment, other relevant immune cell populations—including macrophages, dendritic cells, and NK cells—were not formally evaluated. Furthermore, the absence of quantitative, spatially resolved cytokine profiling in the tumor microenvironment imposes constraints on the mechanistic interpretation and precludes the identification of direct translational connections between *in vitro* and *in vivo* data. Future studies that integrate temporal and spatial histological analyses with transcriptomic profiling would enable a more comprehensive characterization of immune dynamics and further elucidate the mechanisms through which CY12-RP2 potentiates antitumor immunity. Moreover, the functional consequences of PDPN targeting observed in our study, such as changes in immune cell infiltration or cytokine profiles, likely stem from the disruption of this critical CAF-mediated immunosuppressive axis, in addition to any direct effects on tumor cell signaling. Disentangling the relative contributions of tumor cell-derived versus stromal cell-derived PDPN represents a vital direction for future research, as it would refine therapeutic strategies aimed at the PDPN pathway to more precisely overcome microenvironment-driven mechanisms.

In conclusion, our findings define podoplanin (PDPN) as a central mediator of tumor immune escape in melanoma, promoting PD-L1 upregulation in tumor cells via activation of the Wnt/β-catenin signaling axis. Pharmacologic targeting of this PDPN–β-catenin–PD-L1 pathway with the inhibitory peptide CY12-RP2 disrupts PDPN-driven immunosuppression within the tumor microenvironment, positioning this approach as a promising strategy for melanoma therapy. These results establish a rational foundation for subsequent research focused on circumventing immune checkpoint resistance, particularly resistance linked to PD-L1, and advancing personalized immunotherapeutic interventions for patients with melanoma.

## Data Availability

The RNA-seq data generated in this study are available at the NCBI SRA database under the accession number PRJNA1389869. Publicly available datasets were also analyzed, including data from The Cancer Genome Atlas (available at: http://www.ncbi.nlm.nih.gov/geo/, accession GSE65904) and databases such as TISIDB (http://cis.hku.hk/TISIDB/index.php), STRING (https://cn.string-db.org/), and TIMER2.0 (http://timer.cistrome.org/).
