# Correction: Targeting PDPN enhances antitumor T-cell activity by disrupting β-catenin-mediated PD-L1 expression in melanoma

**DOI:** 10.3389/fimmu.2026.1950657

**Published:** 2026-09-24

**Authors:** Chunyan Feng, Yueyue Liu, Xinyue Zhong, Zhenzhen Wei, Hongjing Cui, Jianfeng Yi

**Affiliations:** Research Center for the Differentiation and Development of Traditional Chinese Medicine Basic Theory, Institute for Advanced Study, Jiangxi University of Chinese Medicine, Nanchang, Jiangxi, China

**Keywords:** CD8 lymphocytes+, immunotherapy, PD-L1, podoplanin, Wnt/β-catenin

In the published article, there was an error in **Supplementary Data Sheet 2**. An error was found that the lane labels in **Data Sheet 2** do not match the descriptions provided in the legend of **Figure 3F**. The corrected **Supplementary Data Sheet 2** can be found in the **Supplementary Material**.

The original article has been updated.

