# Supplementary material for: Targeting PDPN enhances antitumor T-cell activity by disrupting β-catenin-mediated PD-L1 expression in melanoma
Source: Front Immunol. 2026 Jan 7;16:1692864. doi: 10.3389/fimmu.2025.1692864 (PMC12819673; doi:10.3389/fimmu.2025.1692864)
Supplement: Supplementary file 1 [file DataSheet1.docx]

**Supplementary material**


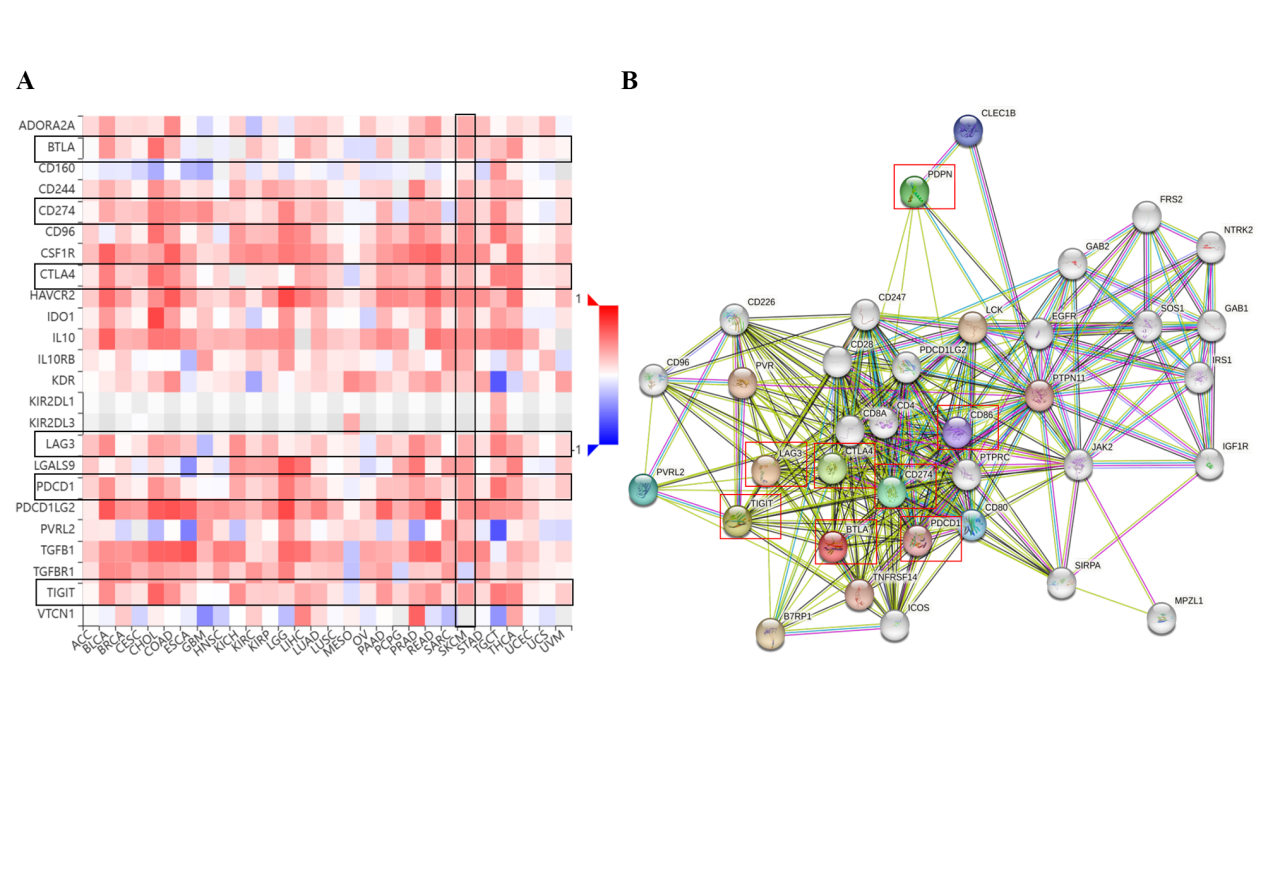


Fig S1: PDPN Correlates with Immunosuppressive Landscapes in Melanoma.

1. Correlation of PDPN and immune checkpoint receptors using TISIDB Database (http://cis.hku.hk/TISIDB/index.php). B. Keyword cluster analysis by VOSviewer.


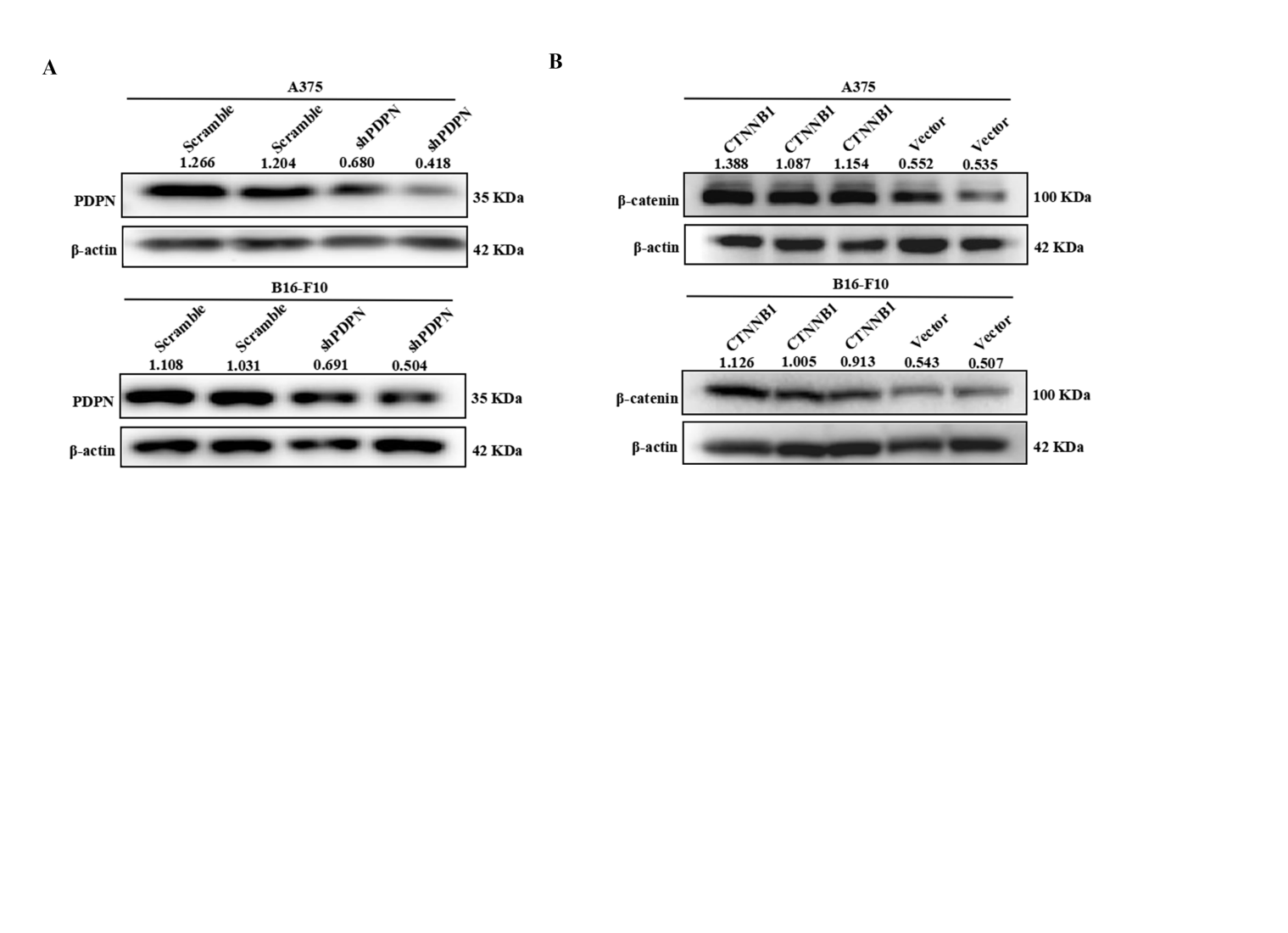


Fig S2: Western blot analysis of protein expression in PDPN-knockdown and CTNNB1-overexpressing A375 and B16-F10 cells.

1. PDPN expression in PDPN-knockdown A375 and B16-F10 cells; B β-catenin expression in CTNNB1-overexpressing A375 and B16-F10 cells.

Table 1Analysis of the correlation between PDPN and immune cell infiltration

| Cancer | CD8+ T  cells | CD4+ T  cells | M1  Macrophage | M2  Macrophage | Treg  cells | NK  cells |
| --- | --- | --- | --- | --- | --- | --- |
| SKCM (n=471) | -2.988 | -0.209 | -5.524 | 6.865 | 1.457 | -2.557 |
| SKCM-Metastasis (n=368) | -2.464 | 0.03 | -5.512 | 6.32 | 1.582 | -2.161 |
| SKCM-Primary (n=103) | -1.488 | -0.05 | -0.563 | 1.691 | -0.768 | -0.468 |

Table 2 Analysis of protein expression characteristics and correlation between PDPN and PD-L1

| Cancer | PDPN-  (≤ normal) | PDPN+  (＞normal) | PD-L1-  (≤ normal) | PD-L1+  (＞normal) | Coexpression |
| --- | --- | --- | --- | --- | --- |
| No tumor (n=1) | 1(100%) | 0 | 1 | 0 | 1(100%) |
| SKCM Primary (n=86) | 33 (38.4%) | 53 (61.6%) | 33 (38.4%) | 53 (61.6%) | 40 (46.5%) |
| SKCM Metastasis (n=16) | 9 (56.2%) | 7 (43.8%) | 2 (12.5%) | 14 (87.5%) | 7 (43.8%) |

Table 3 Analysis of protein expression characteristics and correlation between PDPN and CD8

| Cancer | PDPN-  (≤ normal) | PDPN+  (＞normal) | CD8α-  (≤ MFI 2.0) | CD8α+  (＞MFI 2.0) | Coexpression |
| --- | --- | --- | --- | --- | --- |
| No tumor (n=1) | 1 (100%) | 0 | 0 | 0 | 0 |
| SKCM Primary (n=86) | 33 (38.4%) | 53 (61.6%) | 44 (51.2%) | 42 (48.8%) | 23 (26.7%) |
| SKCM Metastasis (n=16) | 9 (56.2%) | 7 (43.8%) | 14 (87.5%) | 2 (12.5%) | 0 |

Table 4 Antibodies applied for western-blotting, immunofluorescence and flow cytometry

| **antibodies** | **sources** | **catalog No** |
| --- | --- | --- |
| Podoplanin Rabbit mAb | ABclonal | A9242 |
| β-Catenin Rabbit pAb | ABclonal | A11932 |
| Phospho-β-Catenin-S552 Rabbit mAb | ABclonal | AP1315 |
| PD-L1/CD274 Rabbit mAb | ABclonal | A19135 |
| APC anti-human/mouse PD-L1 | Biolegend | 124312/393610 |
| PD-L1 (Extracellular Domain Specific)Rabbit Monoclonal Antibody | CST | Cat##86744 |
| ABflo® 488-conjugated secondary antibody | Abclonal | Cat# AS037 |
| Podoplanin Polyclonal antibody | Proteintech | 11629-1-AP |
| PD-L1/CD274 (C-terminal) Polyclonal antibody | Proteintech | 28076-1-AP |
| ABflo® 594-conjugated secondary antibody | ABclonal | Cat# AS039 |
| ABflo® 647-conjugated secondary antibody | ABclonal | Cat# AS060 |
| CD3 Polyclonal antibody | Proteintech | 17617-1-AP |
| CD8a Polyclonal antibody | Proteintech | 29896-1-AP |
| Granzyme B Polyclonal antibody | Proteintech | 13588-1-AP |
| CD8a Monoclonal antibody | Proteintech | 66868-1-Ig |
| β-Actin Rabbit mAb | ABclonal | AC026 |
