# Supplementary figures and images for: Targeting PDPN enhances antitumor T-cell activity by disrupting β-catenin-mediated PD-L1 expression in melanoma
Source: Front Immunol. 2026 Jan 7;16:1692864. doi: 10.3389/fimmu.2025.1692864 (PMC12819673; doi:10.3389/fimmu.2025.1692864)

Supplementary file 2

Original images of Western membranes

For Fig.3D


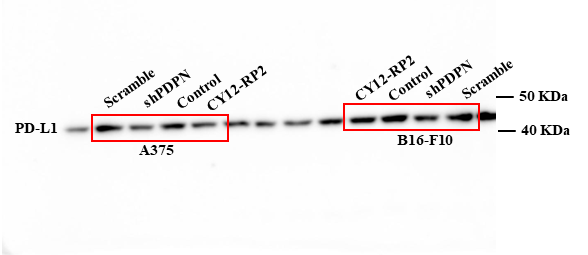


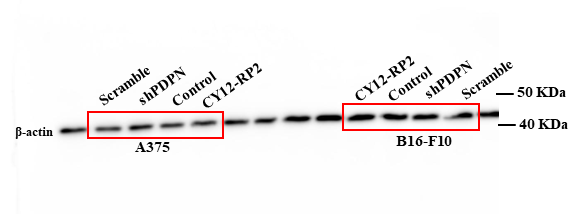


For Fig.3C


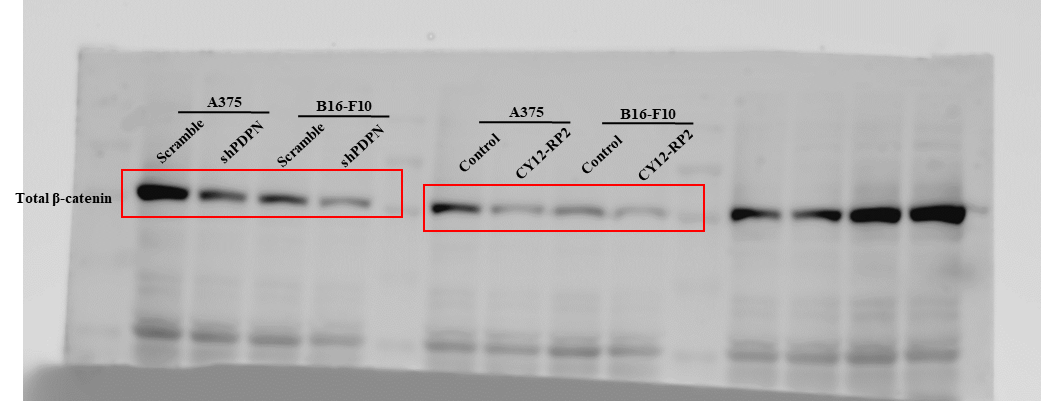


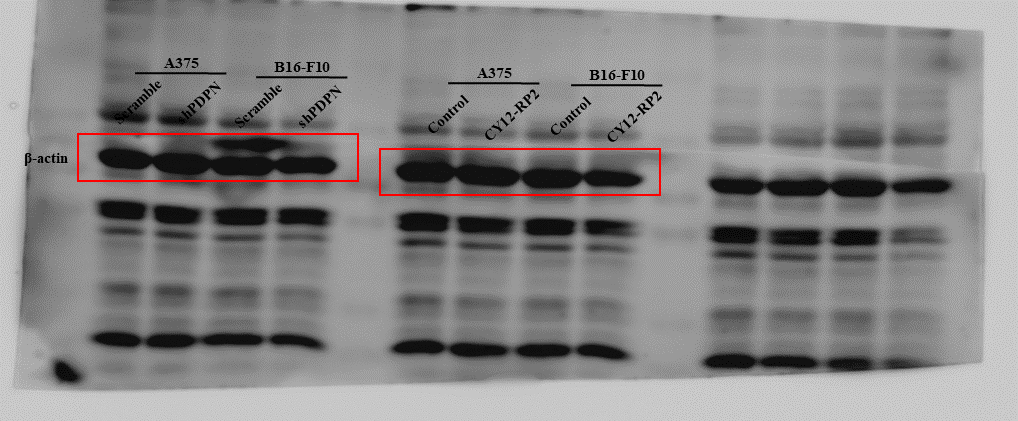


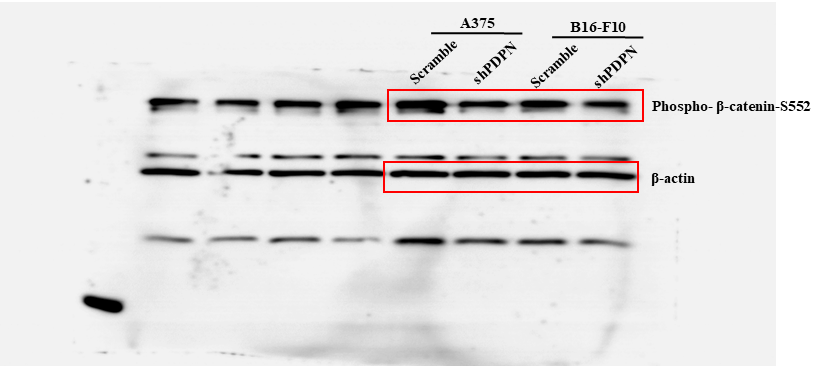


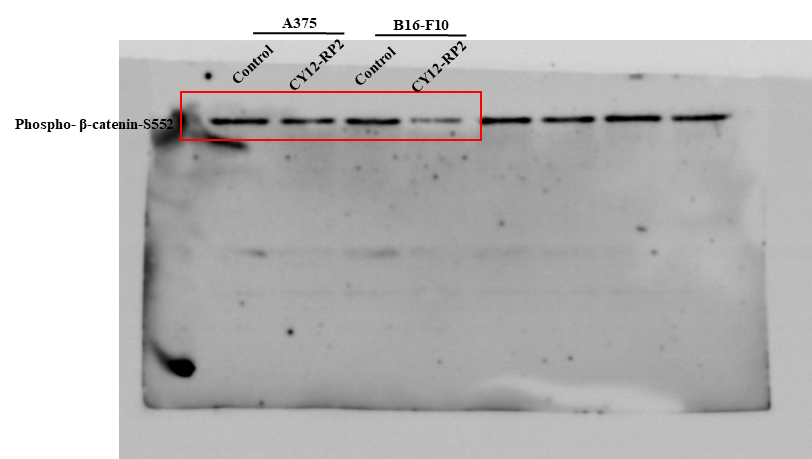


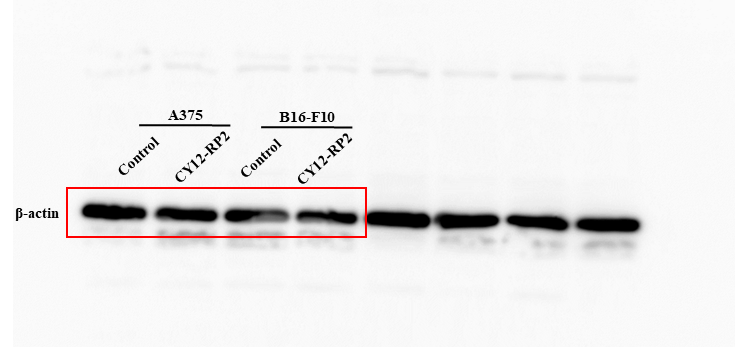


For Fig.3F


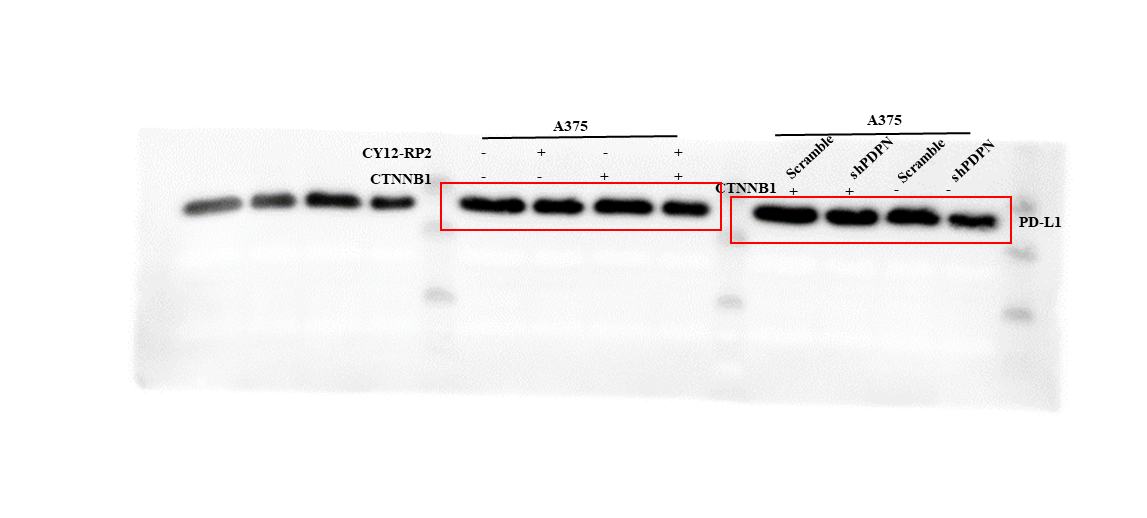


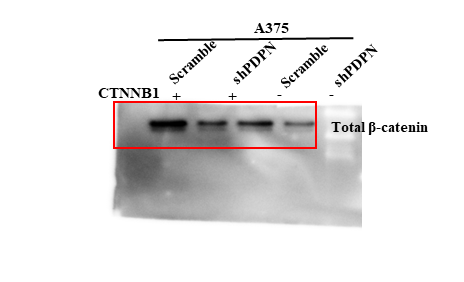


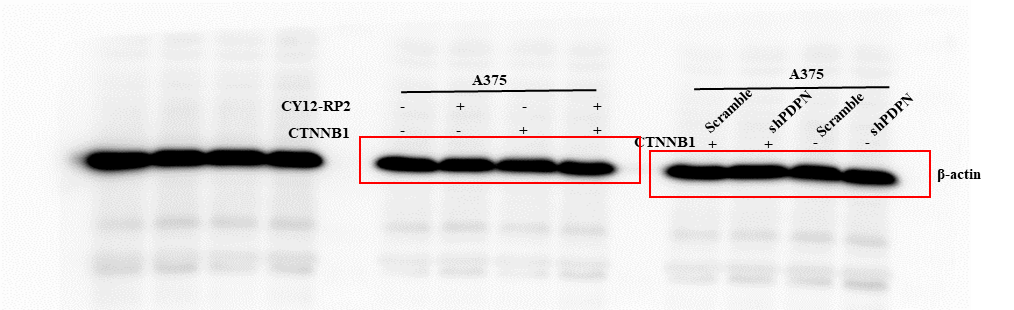


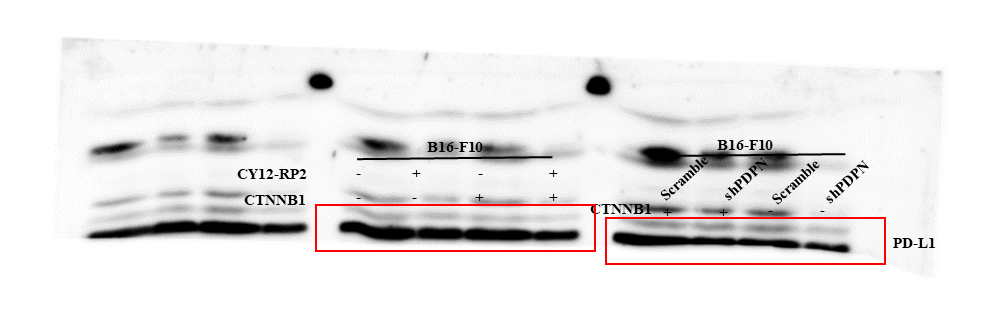


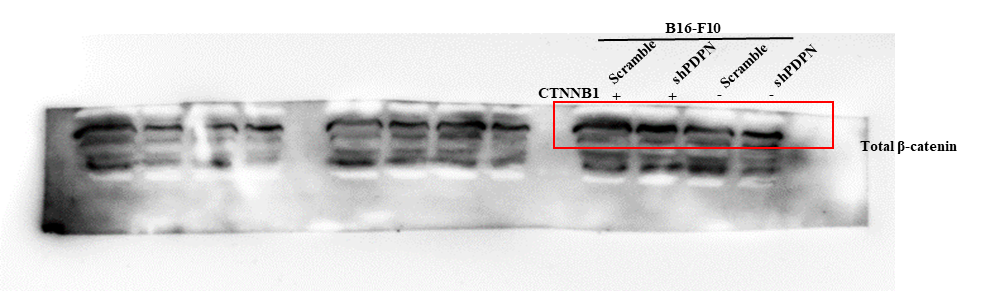


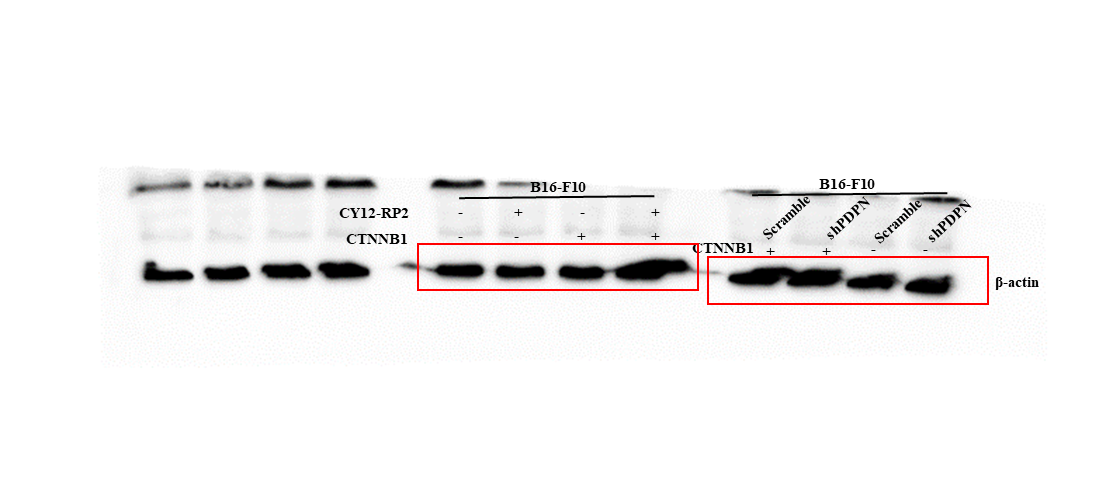


For Fig.S2


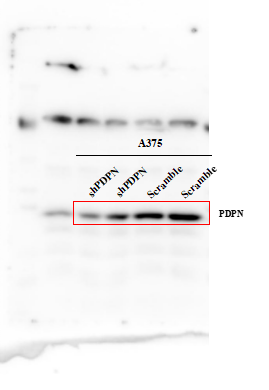

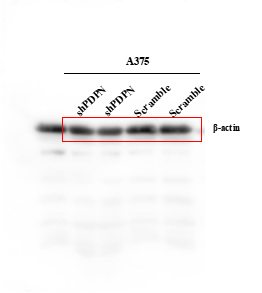


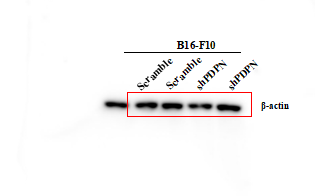

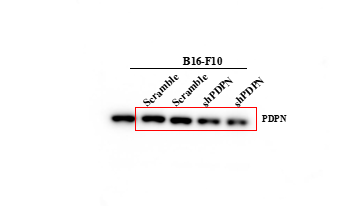


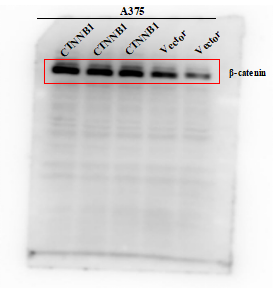

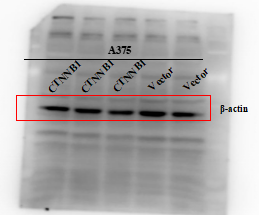


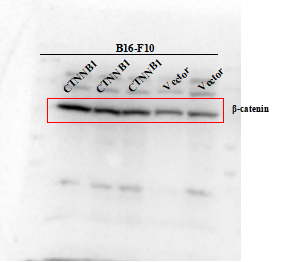

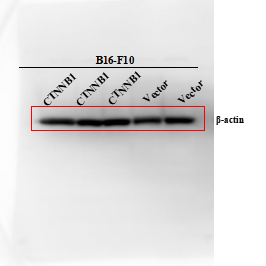

Supplement: Supplementary file 2 [file DataSheet2.docx]
